# Pathogenic or Therapeutic: The Mediating Role of Gut Microbiota in Non-Communicable Diseases

**DOI:** 10.3389/fcimb.2022.906349

**Published:** 2022-07-07

**Authors:** Fan Bu, Xingran Yao, Zhihua Lu, Xiaomin Yuan, Chen Chen, Lu Li, Youran Li, Feng Jiang, Lei Zhu, Guoping Shi, Yugen Chen

**Affiliations:** Department of Colorectal Surgery, The Affiliated Hospital of Nanjing University of Chinese Medicine, Nanjing, China

**Keywords:** gut microbiota, noncommunicable diseases, microbial transmission, fecal microbiota transplantation (FMT), epidemiology

## Abstract

Noncommunicable diseases (NCDs) lead to 41 million deaths every year and account for 71% of all deaths worldwide. Increasing evidence indicates that gut microbiota disorders are closely linked to the occurrence and development of diseases. The gut microbiota, as a potential transmission medium, could play a key role in the transmission and treatment of diseases. The gut microbiota makes noncommunicable diseases communicable. New methods of the prevention and treatment of these diseases could be further explored through the gut microbiota.

## Introduction

Globalization has a long history, but people’s attention to globalization is mostly focused on economic and cultural fields; that is, the extensive and rapid flow of capital, commodities, and personnel has made the entire world a large community. The outbreak of coronavirus 2019 (COVID-19) has given people a deep comprehension of the globalization of the spread of infectious diseases. The COVID-19 pneumonia pandemic that broke out in early 2020 had spread to more than 200 countries and regions as of December 2021, with more than 200 million infections. Infectious diseases, regardless of region, country, culture, or religion, are spreading rapidly around the world, seriously threatening people’s lives and health.

Infectious diseases represent a type of disease caused by various pathogens that can spread from person to person, animal to animal, or person to animal. Diseases spread from animals to people can also be considered infectious diseases. Koch’s postulates are the golden rules for identifying the pathogens of infectious diseases, and these postulates are as follows: the suspected pathogen should be found in individuals with a communicable disease, can be isolated from the host and grown in culture, cause disease when inoculated into a healthy host and can be reisolated from the inoculated host. A report suggests that numerous noncommunicable diseases (NCDs) could have a transmissible microbial component (Finlay, 2020). The definition of NCDs rules out microbial involvement and instead focuses on genetic, environmental, and lifestyle factors. With the deep realization of gut microbes, growing evidence supports that individuals with noncommunicable diseases have intestinal microbiota dysbiosis. As people socialize more frequently, which can multiply the risk of gut microbiota (GM) exchange among individuals in a population, they may promote the spread of NCDs. A recent study examined the faecal and oral microbiota of 287 people from the Fiji Islands, and a subset of spouses could be predicted on the basis of strain-level data alone through core single nucleotide polymorphism (SNP) and flexible genomic region analyses (Brito et al., 2019). This finding illustrates the horizontal transmission of commensal flora in a population and shows that the similarity of the flora can reflect social relationships to a certain extent. Shotgun sequenced dataset analyses of the gut microbiomes of a large cohort of 250 adult twins demonstrated that microbial SNPs are often shared between twins and slowly diverge after the twins live apart, making clear that the behaviour of a family or cohousing affects the similarity of the gut microbiota (Xie et al., 2016). Na Fei et al. isolated one endotoxin-producing bacterium, *Enterobacter cloacae B29*, from the gut of a morbidly obese human and inoculated B29 into germ-free mice on a high-fat diet, inducing obesity and insulin resistance through a single bacterium for the first time, which is also the first case of a human obesity-causing bacterium identified according to Koch’s postulates (Fei and Zhao, 2013).

In traditional beliefs about NCDs, changes in the gut microbiome may be a cause of the diseases, and the disturbed GM contributes to the spread of NCDs. It is worth noting that good microbes may also have a protective effect against NCDs. This review aims to summarize the potentially pathogenic and therapeutic effects of the GM as a vector in a variety of NCDs, to reveal that previously believed noncommunicable diseases have infectious characteristics, and to provide a reference for adjusting the prevention and treatment strategies of NCDs as well as the social behaviours and living habits of populations (**Table 1**).

**Table 1:** The role of gut microbiota as a transmission in different diseases.

Disease	Function	Donor	Recipient animal	medium	Changes after FMT
Obesity	Pathogenic effect	Obese human	Germ-free (GF) C57BL/6J mice	*Enterobacter cloacae B29*	The mice showed obese and insulin resistant with increased endotoxin load and provoked systemic inflammation
Diabetes	Pathogenic effect	db/db male mice	Pseudo-germ-free murine model	Gut microbiota	Bodyweight, fluid intake, food intake, and fasting blood glucose were significantly increased
Diabetes	Pathogenic effect	Sham-operated fa/fa mice	GF Swiss Webster male mice	Gut microbiota	Postprandial peak glucose levels were higher and Alpha diversity was lower than in the RYGB recipients.
Diabetes	Therapeutic effect	RYGB-treated fa/fa mice	GF Swiss Webster male mice	Gut microbiota	Body weight was significantly lower, showing little diabetic phenotype.
Obesity	Pathogenic effect	The obese (Ob) ones of co-twin	GF mice	Gut microbiota	There were significantly greater increases in body mass, adiposity, and metabolism of branched-chain amino acids in the recipient mice.
Obesity	Therapeutic effect	The lean (Ln) ones of co-twin	GF mice	Gut microbiota	Short-chain fatty acids and microbial transformation of bile acid species were increased in the recipient mice.
Obesity	Therapeutic effect	Lean donors	Obese patients with metabolic syndrome	Gut microbiota	There was an improvement in insulin sensitivity, decreased fecal microbial diversity, and an observed increase in plasma metabolites such as GABA in the recipient mice.
Obesity	Therapeutic effect	Obese mice treated with WEGL	Obesity mice of the C57BL/6NCrlBltw genetic lineage through HFD-fed	Gut microbiota	There was less weight gain and fat accumulation, reduced pro-inflammatory cytokine expression in the liver and adipose tissues in the recipient mice.
Obesity	Therapeutic effect	Obese mice treated with HSM	Obesity C57BL/6J male mice through HFD-fed	Gut microbiota	The mice showed less body weight gain, improved intestinal integrity and reduced inflammation and decreased insulin resistance.
Polycystic ovary syndrome (PCOS)	Pathogenic effect	Individuals with PCOS	Antibiotics treated mice	Gut microbiota	The recipient mice displayed insulin resistance, a disrupted estrous cycle and decreased fecal microbial diversity and their ovaries increased numbers of cyst-like follicles
PCOS	Pathogenic effect	Individuals with PCOS	Antibiotics-treated mice	*B. vulgatus*	The recipient mice showed insulin resistance and ovarian dysfunction such as disrupted estrous cycles and ovarian morphology destruction
PCOS	Therapeutic effect	SPF mice	Induced PCOS female SD mice	Gut microbiota /*Lactobacillus*	The recipient mice showed reduced androgen levels, increased serum estrogen levels and improved estrous cycles.
Hypertension	Pathogenic effect	Individuals with primary hypertension	GF mice	Gut microbiota	The recipient mice exhibited significantly higher SBP, DBP, and mean blood pressure (MBP) as compared to controls, as well as elevated heart rate.
Thrombosis	Pathogenic effect	C57BL/6J and NZW/LacJ mice	GF mice	Gut microbiota	Plasma TMAO levels in the C57BL/6J recipient mice were over twice as high as those observed in the NZW/LacJ mice.
Atherosclerosis	Pathogenic effect	C57BL/6J mice	Antibiotic-treated APOE-/- mice	Gut microbiota	The recipient mice had higher plasma TMAO levels and increased atherosclinal plaque burden depend on the choline diet.
Stroke	Therapeutic effect	Young C57BL/6 male mice	Aged C57BL/6 male mice	Gut microbiota	The survival rate of the FTG group increased and restored F: B ratio
Cerebral ischemic stroke	Therapeutic effect	Ischemic mice with decoction treatment	The antibiotics-treated ischemic mice	Gut microbiota rich in SCFAs	The recipient mice had higher SCFAs level and the stroke symptoms were relieved.
Non-alcoholic Fatty liver	Pathogenic effect	Obese human Donor before a dietary weight loss program	GF male C57BL/6J mice	Gut microbiota	PreM group showed a higher level of serum LBP, Ast and concentrations of hepatic triglyceride and total cholesterol, and developed liver macrovesicular steatosis.
Non-alcoholic fatty liver	Therapeutic effect	A genetically Obese human donor after a dietary weight loss program	GF male C57BL/6J mice	Gut microbiota	PostM group showed a lower level of serum LBP, Ast and concentrations of hepatic triglyceride and total cholesterol, and remained normal Morphology.
Liver cirrhosis	Control/Pathogenic effect	Healthy individuals/Patients with HE cirrhosis	GF C57BL/6 mice	Gut microbiota	Cirr-Hum mice had greater neuroinflammation, microglial/glial activation, and GABA signaling and lower synaptic plasticity compared to Ctrl-Hum mice.
Nonalcoholic steatohepatitis(NASH)	Control	Sprague-Dawley mice fed with a control diet	Sprague-Dawley (SD) mice fed with a control diet underwent intestinal decontamination	Gut microbiota	No changes were found.
NASH	Pathogenic effect	SD mice fed with a high-fat diet and high-glucose/fructose syrup (HFGFD)	SD mice fed with a control diet underwent intestinal decontamination	Gut microbiota	The recipient mice presented a reduction of fasting glycemia and HOMA-IR compared with controls and did show a significant increase of intrahepatic P-Akt levels compared to HFGFD-Atr animals indicating a restoration of liver insulin sensitivity after the IM transplantation from healthy individuals.
NASH	Control	SD mice fed with HFGFD	SD mice fed with HFGFD underwent intestinal decontamination	Gut microbiota	No significant changes in the microbiome composition were found.
NASH	Therapeutic effect	SD mice fed with a control diet	SD mice fed with HFGFD underwent intestinal decontamination	Gut microbiota	The mice’s portal hypertension, insulin resistance and endothelial dysfunction reverted and microbial diversity tended to be similar to the control recipients.
Nonalcoholic fatty liver disease (NAFLD)	Therapeutic effect	C57BL/6J male mice treated with quercetin	GF male C57BL/6J mice	Gut microbiota	The recipient mice displayed an opposite pattern to dHFD+ recipients.
NAFLD	Pathogenic effect	C57BL/6J male mice which had a higher response to HFD	GF male C57BL/6J mice	Gut microbiota	The recipient mice displayed an increased BWG, epididymal fat accumulation and impaired insulin sensitivity and showed lower acetate, propionate and butyrate production. They also showed a reduction of *Verrucomicrobia* phyla, *Verrucomicrobiae* class and *Akkermansia* genus.
NAFLD	Therapeutic effect	C57BL/6J male mice	GF male C57BL/6J mice	Gut microbiota	The recipient mice displayed an opposite pattern to dHFD+ recipients and they showed a significant reduction on NLRP3 gene expression.
Hepatic encephalopathy	Therapeutic effect	Eligible healthy donors.	Patients with hepatic encephalopathy	Gut microbiota	Patients who received an FMT had significantly fewer hepatic encephalopathy episodes as well as improved cognitive testing compared with controls.
Chronic constipation	Pathogenic effect	Patients with constipation	Antibiotic depletion mice model.	Gut microbiota	The recipient mice presented a reduction in intestinal peristalsis and abnormal defecation parameters including the frequency of pellet expulsion, fecal weight, and fecal water content.
Slow transit Constipation(STC)	Pathogenic effect	Patients with slow-transit constipation	Pseudo-germ-free mice	Gut microbiota	The recipient mice presented with lower pellet frequency and water percentage, smaller pellet size, delayed gastrointestinal transit time, and weaker spontaneous contractions of colonic smooth muscle.
Diarrhea-predominant IBS	Pathogenic effect	A mouse model of neonatal maternal separation (NMS)	Pseudo-germ-free mice	Gut microbiota	The recipient mice exhibited increased defecation frequency and upregulated bacterial production of intracolonic SCFAs.
STC	Therapeutic effect	Eligible healthy donors.	Patients with slow-transit constipation	Gut microbiota	FMT treatment is more efficient than conventional treatment.
IBS	Pathogenic effect	IBS patients with diarrhea,with or without anxiety.	Germ-free C57BL/6 mice	Gut microbiota	The recipient mice exhibited faster gastrointestinal transit, intestinal barrier dysfunction, innate immune activation, and anxiety-like behavior.
IBS	Therapeutic effect	Eligible healthy donors.	Patients with IBS with diarrhea or with diarrhea and constipation	Gut microbiota	The recipients showed more relief ratio than the control groups.
Inflammatory bowel disease (IBD)	Pathogenic effect	UC and CD patients	Germ-free C57BL/6 mice	Gut microbiota	The recipient mice exhibited decreased GM diversity, alteration of bacterial metabolic functions, and more severe colitis.
DSS colitis	Pathogenic effect	Dss-induced ASC-Deficient colitis mouse models	Adult wild-type mice	Gut microbiota (cohousing)	The recipient mice exhibited increased severity of colitis (more weight loss, higher colitis severity score, and fewer survivals).
IBD	Pathogenic effect	UC and CD patients	Microbiota-depleted IL-10^-/-^ mice with a C57BL/6J background	Gut microbiota	The recipient mice exhibited more pathological inflammation and cytokine expression in the colon and had more *E. faecium*. They showed less increase in weight gain epithelial hyperplasia with lymphoplasmacytosis, obliteration of normal architecture, and erosion.
IBD	Pathogenic effect	UC and CD patients	Microbiota-depleted IL-10^-/-^ mice with a C57BL/6j background	*E.faecium strain ATCC 19434*	The recipient mice exhibited more colitis and colonic cytokine expression. They showed slower increase in body weight, epithelial hyperplasia with lymphoplasmacytosis, obliteration of normal architecture, and erosion.
IBD	Therapeutic effect	Mouse models of colitis treated with anti-mouse IL-1α	GF SAMP mice models of colitis induced by DSS.	Gut microbiota	The recipient mice exhibited reduced mortalit、 decreased bodyweight loss and colonic inflammation and showed a decreased ratio of *Proteobacteria* to *Bacteroidetes*, decreased presence of *Helicobacter* species, and elevated representation of *Mucispirillum* schaedleri and *Lactobacillus salivarius*.
Colorectal Cancer	Pathogenic effect	patients with CRC	GF C57BL/6 mice	Gut microbiota	The recipient mice developed high-grade dysplasia and macroscopic polyps and showed a higher proportion of proliferating (Ki-67-positive) cells and lower richness in microbial compositions.
Major depressive disorder (MDD)	Pathogenic effect	MDD patients	Adult male GF Kunming mice	Gut microbiota	The recipients displayed a decreased center motion distance in the OFT(anxiety-like Behavior)and a decreased proportion of center motion distance in the OFT and an increased duration of immobility in the FST and TST(depression-like behaviors).
Depression	Pathogenic effect/Control	depressed patients	Antibiotic-treated mice	Gut microbiota	The recipient mice showed decreased gut microbiota richness and diversity behavioral and physiological features characteristic of depression including anhedonia and anxiety-like behaviors, as well as alterations in tryptophan metabolism.
Anxiety and depression	Pathogenic effect	Mice that had been exposed to chronic unpredictable mild stress	Antibiotic-treated mice	Gut microbiota	The recipient mice showed higher levels of anxiety- and depression-like behavior compared to the controls and a lower relative abundance of *Lactobacillus* and a higher relative abundance of *Akkermansia.*
Depression	Pathogenic effect	Severe depressive patients	GF mice	Gut microbiota	The recipient mice exhibited anxiety- and depressive-like behaviors.
ASD	Pathogenic effect/Control	ASD patients	GF C57BL/6J mice	Gut microbiota	Mice with FMT from ASD donors showed increased repetitive behavior, decreased sociability, decreased locomotion, and showed different GM composition (increased *Lachnospiraceae* and decreased *Lachnospiraceae*)
Schizophrenia	Pathogenic effect	Drug-free patients with schizophrenia	Antibiotic-treated male C57BL/6J mice	Gut microbiota	The recipient mice displayed behavioral abnormalities such as psychomotor hyperactivity, impaired learning and memory and showed elevation of the kynurenine-kynurenic acid pathway of tryptophan degradation in both periphery and brain, as well as increased basal extracellular dopamine in the prefrontal cortex and 5-hydroxytryptamine in the hippocampus.
Alzheimer’s disease	Pathogenic effect	PD mice	Wild-type male C57BL/6 mice	Gut microbiota	The recipient mice display impaired motor function and decreased striatal dopamine (DA) and serotonin (5-HT) levels.
Alzheimer’s disease	Therapeutic effect	Wild-type male C57BL/6 mice	PD mice	Gut microbiota	The recipient mice showed reduced gut microbial dysbiosis, decreased fecal SCFAs, alleviated physical impairment, and increased striatal DA and 5-HT content, and the activation of microglia, astrocytes in the substantia nigra, and expression of 27 TLR4/TNF-α signaling pathway components in the gut and brain were reduced.
Parkinsonism	Therapeutic effect	Healthy wild-type (WT) mice	Amyloid and neurofibrillary tangles (ADLPAPT) transgenic mouse model of AD	Gut microbiota	The formation of amyloid β plaques and neurofibrillary tangles, glial reactivity, and cognitive impairment were ameliorated. Additionally, the FMT reversed abnormalities in the colonic expression of genes related to intestinal macrophage activity and the circulating blood inflammatory monocytes in the ADLPAPT recipient mice.

## Metabolic Diseases

### Diabetes

Diabetes is a major cause of death and disability worldwide. Disability resulting from diabetes has grown substantially since 1990, with particularly large increases among people aged 15-69 years (Krug, 2016). People with all types of diabetes are at risk of developing a range of complications that can affect their quality of life greatly, and this disease leads to high costs of medical care.

It has been found that the GM can modulate inflammation, gut permeability, glucose metabolism and fatty acid oxidation, and combined with the effects of bacteria, can participate in regulating the metabolism of diabetes (Gurung et al., 2020). In a recent study, Yu, F., et al. used C57 BLKS db/db male mice as a model, and they found that the β-diversity and relative abundances of gut bacteria were altered in db/db mice (Yu et al., 2019). Furthermore, GM from db/db mice transplanted into pseudogerm-free mice showed a significant change in metabolic parameters. The findings suggest that the GM of diabetic mice has transmissible components and that the transmission of abnormal GM might contribute to the development of type 2 diabetes mellitus (T2DM). A meta-analysis conducted an in-depth study of 75,498 couples diagnosed with diabetes by evaluating the results of 6 studies (Leong et al., 2014). The conclusion was that the diagnosis of diabetes in one spouse increased the risk of diabetes in the other spouse by 26%. The study excluded genetic factors associated with kinship, and the findings suggest that the transmission of gut microbiota between spouses may be a risk factor for diabetes, but it is difficult to isolate the effects of gut microbiota from those of dietary and environmental factors.

Metformin is the first-line medication for diabetes, and its beneficial effect on glucose metabolism may be mediated by the GM (Forslund et al., 2015). A recent study shows that metformin may enhance intestinal barrier function and protect intestinal integrity by regulating intestinal bacterial composition, thereby reducing the absorption of lipopolysaccharide. Roux-en-Y gastric bypass (RYGB) is an effective surgical method for type 2 diabetes (Zhou et al., 2016). A study revealed that RYGB-treated fa/fa mice exhibited greater microbiota diversity in the ileum, and their microbiota composition resembled that of nondiabetic fa+ mice (Arora et al., 2017). The disease phenotype of diabetes was not transferred by the transplantation of microbiota from RYGB-treated fa/fa mice to GF mice. In contrast, postprandial peak glucose levels were higher in mice that received gut microbiota from sham-operated mice. From these studies, we speculate that the GM may be an important target of drugs and surgical treatment for diabetes.

### Obesity

Both obesity and diabetes are metabolic diseases, between which the interaction mechanism is still unclear, but both patients with obesity and patients with diabetes have insulin resistance and mild inflammatory reactions. Obesity is not only an independent disease but also an important risk factor for many chronic diseases, such as nonalcoholic fatty liver disease (NAFLD), cardiovascular and cerebrovascular diseases, and hypertension. There is growing evidence that changes in the gut microbiota are one of the causes of obesity. A mouse-based study (Cox et al., 2014) showed that early administration of antibiotics altered the gut microbes of mice, increasing mouse fat mass and making mice prone to obesity, and similarly, early use of antibiotics also increased the risk of obesity in humans (Podolsky, 2017).

**Figure 1:**
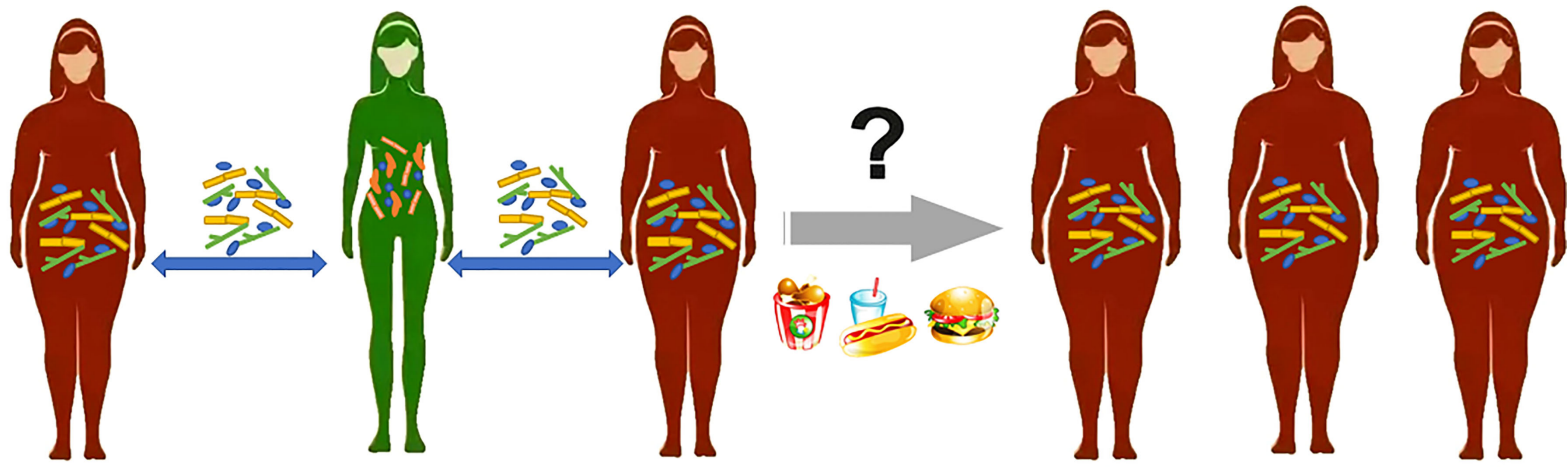
A person’s chances of becoming obese increased by 57% if he or she had a friend who became obese in a given interval. Gut microbiota between friends may be a risk factor for obesity, but it is difficult to isolate from dietary and environmental interference.

Studies have found that the transplantation of GM isolated from genetically susceptible or diet-induced obesity animal models into GF animals will cause significant weight gain, indicating that the symptoms of obesity can be transmitted through the GM. In a faecal microbiota transplantation (FMT) experiment, GF mice were found to have gained weight and showed the obesity metabolic phenotype after FMT from an obese twin (Ridaura et al., 2013). Researchers found that cohousing Ln and Ob mice (mice with faecal microbiota from an obese and lean cotwin, respectively) prevented the development of increased adiposity and body mass in Ob cage mates and suppressed the obesity metabolic phenotype. Phenotypic changes in cage-housed mice provide further evidence of the important role of the gut microbiota in the spread of obesity, which may also be contagious in humans. In research on a densely interconnected social network of 12,067 people, a person’s chances of becoming obese increased by 57% if he or she had a friend who became obese in a given interval (**Figure 1**). If one spouse became obese, the likelihood that the other spouse would become obese increased by 37% (Christakis and Fowler, 2007). Although this paper does not discuss the microbiota as a vector for increasing the incidence of obesity in social networks, the internal mechanism of this phenomenon deserves our in-depth study.

In terms of disease treatment, a clinical trial included 38 obese patients for FMT therapy, among whom insulin sensitivity was significantly improved at 6 weeks after lean-donor FMT, accompanied by observed changes in plasma metabolites such as γ-aminobutyric acid (GABA) (Kootte et al., 2017). In addition to the transplantation of lean GM, animal experiments have shown that the transplantation of the gut microbiota of obese mice treated with Chinese medicine, such as Ganoderma lucidum mycelium (WEGL) and Hirsutella Sinensis (HSM), can also reduce the obesity characteristics of obese mice and restore their phenotype to normal, proving that the drugs can alleviate the disease by improving the GM of obese mice and that the GM can continue to play a therapeutic role in obese mice (Chang et al., 2015; Wu et al., 2018). This kind of benign GM transmission is also worthy of further research, which may provide a new method for the treatment of metabolic diseases.

### Polycystic Ovary Syndrome

The occurrence of PCOS is affected by factors such as genetic, neuroendocrine, metabolic, environmental, and lifestyle factors (Delitala et al., 2017). Several recent studies have shown that there are GM disorders in PCOS animal models and women with PCOS (Insenser et al., 2018; Sherman et al., 2018). The possible mechanism is related to the activation of the immune system and the stimulation of the secretion of gut-brain peptides (Yurtdas and Akdevelioglu, 2020).

In a recent study, stools were transplanted into mice by gavage from healthy controls or patients with PCOS (Qi et al., 2019). Compared with mice transplanted with stools from healthy controls, mice transplanted with stools from individuals with PCOS displayed insulin resistance and disrupted oestrous cycles, and their ovaries showed increased numbers of cyst-like follicles and fewer corpora lutea. The researchers further gavaged wild-type mice with *Bacteroides vulgatus*(*B. vulgatus)*, which is highly expressed in patients with PCOS, and found that the recipient mice also showed insulin resistance and ovarian dysfunction. In another study researchers treated PCOS mice with *Lactobacillus* and faeces from healthy mice, finding that both groups showed reduced androgen levels and PCOS mice showed increased serum oestrogen levels and improved oestrous cycles (Guo et al., 2016). FMT was more effective in elevating oestradiol and estrone levels and improving oestrous cycles than *Lactobacillus* transplantation.

These studies have shown that PCOS can be spread through the transplantation of the whole GM or individual pathogenic bacteria, and normal GM and beneficial strains can also be transplanted into PCOS patients for treatment. The GM plays a vital role in regulating host immunity and affecting hormone secretion in PCOS. The results of these studies may help us prevent PCOS by cutting off the transmission pathway of flora in clinical use and contribute to further studies on the clinical feasibility of FMT in the treatment of PCOS. The distribution of the GM and the changes in the abundance of different genera may play key roles in the occurrence and development of metabolic diseases. Disease phenotypes can be replicated by FMT from diseased animals or humans. More studies are needed to confirm the relationship between the occurrence of disease and the degree of social intimacy between people.

## Cardiovascular Disease

With the increasing ageing of the population in China, cardiovascular disease has become one of the leading causes of death. As previously mentioned, microbes may influence the occurrence of obesity and type 2 diabetes, both of which can contribute to cardiovascular disease. Metabolites produced by the GM, such as short-chain fatty acids and secondary bile acids, influence the incidence of cardiovascular disease. These metabolites can also have direct effects on the formation of atherosclerotic plaques by causing chronic inflammation and affecting endothelial cell function and trimethylamine N-oxide levels (Komaroff, 2018).

In a recent study, at 10 weeks after the transplantation of GM from individuals with primary hypertension, the recipient mice exhibited significantly higher systolic blood pressure, diastolic blood pressure, and mean blood pressure (MBP) than controls, as well as elevated heart rate, suggesting that increased blood pressure can be caused by GM transfer (Li et al., 2017). Researchers from the Cleveland Clinic transplanted GM from C57BL/6J (a high (trimethylamine N-oxide) TMAO-producing atherosclerotic disease-prone strain) and NZW/LacJ (a low TMAO-producing disease-resistant strain) mice into GF mice. The plasma TMAO levels in the C57BL/6J recipient mice were over twice as high as those observed in the NZW/LacJ recipient mice. Moreover, following microbial transplantation with C57BL/6J caecal contents on a choline-supplemented diet, recipient mice showed a 10-fold increase in plasma TMAO levels and an accompanying significant shortening of time to blood flow cessation during an *in vivo* thrombosis assay, suggesting that thrombosis potential can be transmitted to GF mice through the flora (Zhu et al., 2016). In another study, C57BL/6J and NZW/LacJ mice were selected as donors for faecal microbial transplantation into antibiotic-treated APOE-/- mice, and similar to the above results, mice receiving faeces from C57BL6J mice had higher plasma TMAO levels and increased atherosclerotic plaque burden depending on the choline diet (Gregory et al., 2015). In addition to direct transplantation, Brandsma, E., et al. treated Ldlr^-/-^ mice with antibiotics and subsequently transplanted them with faecal microbiota from Casp1^-/-^ mice based on a cohousing approach, and the proinflammatory flora was transferred between the two groups, accelerating the atherosclerosis process in Ldlr^-/-^mice (Brandsma et al., 2019). The findings of these studies suggest that the transmission of undesirable microbiota can contribute to disease in susceptible animals.

The GM is closely related to the occurrence of CVD through multiple pathways. The gut microbiota may act as a vector to cause the transmission of hypertension, atherosclerosis, and thrombosis among animals, and FMT may be one of the treatments for CVD. Spychala, M.S., et al. made aged male mice have a young microbiota through faecal transplant gavage (FTG), and mice were subjected to ischaemic stroke (MCAO) through surgery; the survival rate of the FTG group increased to that of the controls, and their F:B ratios were restored (Spychala et al., 2018). A growing number of studies have taken the ability of bacteria to produce trimethylamine (TMA) as a target, developing nonlethal inhibitory drugs targeting the GM to reduce the occurrence of CVD by reducing the levels of TMA and TMAO (Roberts et al., 2018). Meldonium showed a beneficial effect on TMAO concentrations through altering TMA-TMAO gut microbiota-dependent production (Dambrova et al., 2013). Rhizoma coptidis (RC) is the dried rhizome of a medicinal plant from the family Ranunculaceae and contains alkaloids such as berberine, coptisine, and palmatine (He et al., 2016). Feeding of RC alkaloids to hyperlipidemic mice significantly changed the gut microbiota and the bile acid pathway to reduce levels of triglycerides, total serum cholesterol, LDL cholesterol, lipopolysaccharide, and total bile acids in mice (Wu and Tan, 2019). Another study showed Persimmon tannin as a type of condensed tannin highly polymerized which non-absorbed in the intestine. The anti-hyperlipidemic and cholesterol-lowering effects of this compound on high-cholesterol diets fed SD rats were partly mediated by altered gut microbiota composition (Zhu et al., 2018). The idea of drug research targeting specific functions of GM can also be applied to other GM-related diseases.

## Liver Disease

The liver is anatomically connected to the intestine through the portal vein. Most of the blood supply of the liver comes from the intestine through the portal vein. The bacteria and their metabolites in the intestine flow back to the liver through the portal vein. Proinflammatory changes in the liver and intestine are thus mediated. Proinflammatory changes in the liver and intestine mediate the development of liver fibrosis, cirrhosis, and hepatocellular carcinoma (Tripathi et al., 2018).

A study confirmed that the GM from obese mice before weight loss can cause mice to develop fatty liver by transplanting GF mice with a different GM from a genetically obese human donor before and after a dietary weight loss programme (Wang et al., 2018). A disordered GM may inhibit the expression of peroxisome proliferator-activated receptor alpha (PPARα) and thereby inhibit the genes associated with lipid decomposition, further leading to steatosis. In different stages of liver disease progression, the number and distribution of gut microbes change. The latest study induced liver cirrhosis in GF mice through FMT from healthy individuals or patients with cirrhosis (Liu et al., 2020). Mice with FME from individuals with cirrhosis had higher neuroinflammation, microglial/glial activation, GABA signalling, and GM dysbiosis than mice with FMT from healthy individuals, which reveals that the gut-liver-brain axis plays an important regulatory role in liver disease.

There is increasing evidence that the GM is the key to steatosis, inflammation and even fibrosis in various aetiology models of liver disease (Le Roy et al., 2013; Schnabl and Brenner, 2014; Jiang et al., 2015). A study showed that the transplantation of a healthy GM into high-fat diet mice can reverse portal hypertension, which is one of the early manifestations of nonalcoholic steatohepatitis (NASH) (Garcia-Lezana et al., 2018). The influence of the GM on portal hypertension may be mediated by the regulation of liver endothelial function. In a new study published by Molecular Nutrition and Food Research, through the transplantation of GM into GF mice, it was found that recipient mice transplanted with the GM of high quercetin-responsive donor mice exhibited a protective phenotype against high-fat diet (HFD)-induced nonalcoholic fatty liver disease (NAFLD), which is closely related to *Akkermansia* bacteria in the intestine (Porras et al., 2019).

Hepatic encephalopathy is a common complication of end-stage liver disease that can be alleviated by regulating the GM. Compared with healthy individuals, patients with hepatic encephalopathy had fewer short-chain fatty acid-producing bacteria and more potentially pathogenic Enterobacter sp. In a clinical trial of FMT for hepatic encephalopathy, patients receiving FMT had significantly fewer hepatic encephalopathy episodes and improved cognitive test scores, with efficacy maintained for up to 12 months, compared to the control group (Allegretti et al., 2019). In another study, FMT was performed for recurrent hepatic encephalopathy (Bajaj et al., 2019). Compared with the standard management group, the cognitive ability of patients in the FMT group was improved, no hepatic encephalopathy occurred within 5 months of follow-up, and the hospitalization rate was reduced. In both trials, FMT therapy significantly improved patients’ cognitive ability and reduced the recurrence rate of hepatic encephalopathy. Although there was no significant increase in adverse events, the potential effect of microbial transplantation on advanced liver disease still needs a large number of clinical trials for verification.

The GM plays a vital regulatory role in the gut-liver-brain axis, the disorder of which accelerates the development of liver diseases in many ways. FMT may be effective in slowing down the progression of liver diseases and treating hepatic encephalopathy.

## Intestinal Diseases

The intestinal tract is an environment where a large number of microorganisms live, and intestinal diseases have the most direct relationship with the GM. The vector roles of flora in different intestinal diseases are discussed below.

### Functional Bowel Disease

Functional gastrointestinal diseases (FGIDs) are a common clinical disease type, most of which involve mainly patients’ symptoms instead of changes in gastrointestinal structure. The Rome IV criteria refer to FGIDs as disorders of gut-brain interaction (DGBIs), and irritable bowel syndrome (IBS) and functional constipation (FC) are the most common DGBIs of the lower gastrointestinal (GI) tract.

A study transplanted GM from patients with chronic constipation into antibiotic-cleaned mouse models (Cao et al., 2017). The results showed weakened intestinal peristalsis, abnormal defecation parameters, and decreased faecal water content after GM transplantation from patients with constipation. Serotonin transporter (SERT) levels in intestinal tissue were obviously upregulated, the content of 5-hydroxytryptamine (5-HT) was decreased, and gastrointestinal motility function was destroyed. GM imbalance may weaken the contraction of intestinal muscles by increasing the expression of SERT, which leads to constipation. The findings from another study also suggested that FMT can reproduce the clinical phenotype of patients with constipation in GF mice, the mechanism of which may be that FMT affects intestinal gastrointestinal motility by changing the metabolites derived from microorganisms (Ge et al., 2017). However, gut motility is regulated by multiple signalling pathways, and the intestinal microbiota might be just one of the causes.

As a transmission medium, the GM can not only replicate the clinical phenotype of constipation but also be administered for constipation treatment. A randomized controlled trial (RCT) study from China included 60 adult patients with slow-transit constipation (STC) (Tian et al., 2017). The control group received traditional treatment, such as education, behavioural strategies, and oral laxatives, while the intervention group was additionally provided 6 days of FMT through endoscopy. Compared with the control group, the intervention group showed better clinical remission and cure rates. However, the intervention group reported more adverse events than the control group, which were mainly related to the endoscopic operation.

Irritable bowel syndrome (IBS) is a group of intestinal dysfunction diseases with continuous or intermittent attacks characterized by abdominal pain, abdominal distension, abnormal defecation habits, and/or changes in stool characteristics. IBS is a symptom cluster resulting from diverse pathologies, and GM imbalance may be the initial factor of IBS.

De Palma, G., et al. colonized germ-free mice with the faecal microbiota from healthy control individuals or IBS patients with diarrhoea (IBS-D) to evaluate a functional role for commensal gut bacteria in IBS (De Palma et al., 2017). Mice receiving the IBS-D faecal microbiota, but not mice receiving the healthy control faecal microbiota, exhibited faster gastrointestinal transit, intestinal barrier dysfunction, innate immune activation, and anxiety-like behaviour. In addition, mice receiving flora from patients with moderate anxiety showed anxiety-like behaviour. This result indicates that in addition to IBS itself, the depression accompanying IBS can also be transferred to GF mice by GM transplantation. This finding will be discussed in the section on mental illness. A double-blind, randomized, placebo-controlled trial included 83 patients with moderate to severe IBS, and the symptoms of 65% of the patients were significantly relieved after receiving FMT treatment for 3 months, while the symptoms of only 43% of the patients in the placebo group were relieved, but this effect was difficult to maintain for 12 months (Johnsen et al., 2018). The latest meta-analysis included 4 RCT studies with a total of 254 participants. Compared with the placebo treatment group, there was no significant difference in the overall improvement of IBS symptoms in the FMT group at 12 weeks. The heterogeneity between studies was significant; therefore, conclusions that FMT can improve IBS symptoms cannot be made.

### Inflammatory Bowel Diseases

Inflammatory bowel diseases include ulcerative colitis (UC) and Crohn’s disease (CD). Most people think that the pathogenesis of IBDs is caused by the combined action of mucosal barrier function, gut microbiota, and mucosal immunity dysfunction. Studies showed that the richness and diversity of the GM in IBD patients were decreased, beneficial bacteria such as Bifidobacterium and Lactobacillus were reduced, and pathogenic bacteria such as adhering invasive *Escherichia coli*, *Clostridium difficile*, and avian *Mycobacterium subspecies paratuberculosis* were increased (Chassaing and Darfeuille-Michaud, 2011).

A study transplanted GM from IBD patients to germ-free mice, and the results showed that the GM diversity of mice was reduced and the metabolic function of bacteria was changed (Nagao-Kitamoto et al., 2016). The microbiota also promoted the development of colitis when used to colonize IBS-prone interleukin 10-deficient mice. Another experiment demonstrated the vector effect of the gut microbiota on the transmission of IBD through cohousing experiments. Raising ASC^-/-^ mice with newborn or adult wild-type mice in the same cage can transfer the pathogenicity of the microbiota and lead to the exacerbation of DSS-induced colitis by inducing cytokines (Elinav et al., 2011). In the intestine, NLRP6 protects mice against dextran sulfate sodium-inducing epithelial cell injury. Mechanistically, NLRP6^-/-^ mice were shown to display impaired production of IL-18, a cytokine important for epithelial barrier repair. However, subsequent studies showed that Nlrp6 does not shape gut microbial ecology and does not alter DSS colitis susceptibility in animal facilities when controlling for nongenetic confounders. This finding dismisses the suggested role for inflammasomes in controlling host health through the regulation of intestinal ecology (Lemire et al., 2017). Mamantopoulos et al. suspect that prior observations were not genetically imposed but rather represented legacy effects resulting from differential maternal inheritance and/or long-term separate housing (Mamantopoulos et al., 2017). A recent study performed a comparative analysis of the GM of IBD patients and healthy people and found that *Enterococcus faecium* was the most different species. (Seishima et al., 2019). Compared with the *Enterococcus faecium* strain isolated from the faeces of healthy humans, the *Enterococcus faecium* strain isolated from the faeces of UC patients promoted the pathological score of colitis and the expression of inflammatory factors in colitis-susceptible IL10^−/−^ mice. The results above indicated that the whole GM or specific harmful strains of infected animals or patients with IBD could cause the normal mice to contract the disease or aggravate the disease of susceptible mice. In the population, the spouses of IBD patients often have a similar GM disorder, and the disease incidence is higher than accidental, but the incidence is e still affected by many factors, such as genetics and environment. An epidemiological study on IBD revealed that the corresponding figures for UC were estimated at 16% for monozygotic twins and 4% for dizygotic twins, suggesting a weaker heritable component for this disease (Ananthakrishnan, 2015).

Research on geography and immigration has shown that countries in Europe and North America are high-UC-incidence countries, and countries in Southeast Asia, such as India and Japan, are low-UC-incidence countries. This research also identified the incidence of UC in first-generation and second-generation Indian migrants to the UK to be higher than the incidence in residents of the countries of origin, and the increased risk was most pronounced in the second-generation migrants, similar to the native UK population. This finding also indicates the importance of environmental factors for the occurrence of UC; additionally, changes in dietary habits and social life make the transmission of GM among people more frequent, which may be one of the mechanisms causing the spread of IBDs. In another study, DSS-induced colitis mice were kept in cages with healthy littermates, and it was found that the GM of mice with colitis tended to be normalized, their intestinal epithelial barrier was restored, and their colitis was ameliorated (Spalinger et al., 2019). However, neither colitis mice nor healthy mice without the PTPN22 gene (a known risk gene for several chronic inflammatory diseases) showed improved symptoms with cohousing. Ge Hong, an ancient Chinese physician, applied faecal suspensions for the treatment of severe diarrhoea for the first time (Drew, 2016). Currently, an increasing number of doctors perform standard FMT therapies for the treatment of IBDs. A meta-analysis included 31 studies of FMT for the treatment of IBD, and the results showed that the final remission rates of UC and CD were 39.6% and 47.5%, respectively, and the overall adverse event rate was less than 1% (Lai et al., 2019).

## Colorectal Cancer

Colorectal cancer (CRC) is the fourth most lethal cancer in the world. Anticancer therapies, including immunotherapy, have relatively poor efficacy in CRC. Colorectal cancer is the result of the synergistic effect of many factors, such as the environment, eating habits, and genetics. As GM research has increased, increasing evidence has shown that the GM is closely related to the occurrence and development of CRC. A study showed that the GM may be the driving factor for CRC development and proposed a “driver-passenger” pattern. First, the original intestinal bacteria (driving factor) attack the intestine, driving DNA damage to cause CRC. Second, the occurrence of tumours will lead to changes in the intestinal microecology that are more conducive to the proliferation of conditional bacteria (bacterial passengers). (Marchesi et al., 2016).

A study transplanted GM from CRC patients and healthy humans into antibiotic-treated mice, and the mice were moulded with AOM (Wong et al., 2017). The results showed that significantly higher proportions of conventional mice fed stools from individuals with CRC than mice fed stools from healthy controls developed high-grade dysplasia (*P*<0.05) and macroscopic polyps (*P*<0.01). In addition, the results of real-time PCR revealed the upregulation of genes involved in cell proliferation, stemness, apoptosis, angiogenesis, invasiveness, and metastasis in mice fed stools from CRC patients. Compared with healthy individuals, there are specific gene promoters in individuals with CRC, including Wif1, PENK, and NPY. The cumulative methylation index (CMI) of these gene promoters in individuals with CRC has been found to be significantly higher than that in healthy controls. Studies have shown that the transplantation of GM from CRC mice into germ-free mice can induce a higher number of hypermethylated genes in murine colonic mucosa (Sobhani et al., 2019). Thus, CRC-related dysbiosis induces the methylation of host genes, and corresponding CMIs together with associated bacteria are potential biomarkers for CRC.

The interaction mechanism between intestinal diseases and the GM is complex and involves aspects such as immune activation, intestinal mucosal damage, and inflammatory cytokine release. Intestinal diseases involved in GM disorders are more likely to be transmitted through the faecal-oral route; therefore, people should pay more attention to personal hygiene habits to prevent the occurrence of intestinal diseases. The application of FMT therapy in IBS/IBDs is also gradually developing, and its safety and effectiveness still need further clinical data verification.

## Mental Illness

With the widespread analysis of the changes in germ-free animals and their microbiota induced by exposure to probiotics, antibiotics, and FMT, researchers have discovered that there are multiple complex interaction pathways involved in the microbiota-gut-brain axis. Changes in the GM and its metabolites can affect the development and function of the central nervous system through the brain-gut axis.

Compared with normal mice, germ-free mice showed higher adrenocorticotropic hormone (ACTH) release after stimulation, which could be alleviated by early colonization of the GM. This result shows that the GM has a profound influence on the activity of the hypothalamic–pituitary–adrenal (HPA) axis (Sudo et al., 2004). Neurotransmitters produced by the GM, such as 5-HT, tryptophan, dopamine and GABA, also affect brain function (Bravo et al., 2011). For example, GABA is an important inhibitory neurotransmitter in the central nervous system (CNS) and plays an important role in the cerebral cortex, hippocampus, thalamus, basal ganglia and cerebellum. When the body lacks GABA, anxiety, restlessness, fatigue, and other negative emotions result. A study showed that *Bifidobacterium* and *L. rhamnosus* strains can produce GABA, and GM disorders may change GABA levels (Strandwitz et al., 2019). There is increasing evidence that the occurrence of phenotypic depression, autism, Alzheimer’s disease and other mental illnesses is closely related to disorders of the microbiota-gut-brain axis (Sharon et al., 2016).

## Depression

With the increasing pressures of life and work, the incidence of depression is on the rise worldwide, the aetiology of which involves neuroendocrine, immune system, metabolic and neurotransmitter disorders. Data from animal studies have suggested that GM imbalance affects the occurrence and development of depression through increased immune activation, reduced tryptophan metabolism and HPA axis dysregulation. A study showed that the abundance of Firmicutes, Actinobacteria, and Bacteroidetes in the GM of patients with major depressive disorder (MDD) was significantly increased (Zheng et al., 2016). FMT of GF mice with ‘depression microbiota’ derived from MDD patients resulted in depression-like behaviours compared with colonization with ‘healthy microbiota’ derived from healthy control individuals. Another study also reproduced the behaviour and pathological characteristics of depression by transplanting GM from depressed patients to antibiotic-treated mice, and the GM in depressed patients may induce the occurrence of depression by regulating the metabolism of tryptophan in mice (Kelly et al., 2016). A study showed that the absence of a GM can induce antianxiety- and antidepressant-like behaviours in GF mice, while those transplanted with GM from CUMS mice exhibit anxiety-like and depression-like behaviour (Luo et al., 2018). Glucocorticoid receptor pathway genes are downregulated in mice with anxiety- and depression-like phenotypes. GM imbalance may lead to abnormal behaviour in mice through the glucocorticoid receptor pathway.

In terms of treatment, a systematic review evaluated 21 studies, and the findings suggested that FMT can affect the symptoms of psychiatric disorders (Chinna et al., 2020). These findings were shown for both the relief of psychiatric symptoms resulting from the transfer of microbiota from healthy donors to ill recipients and the transmission of symptoms through the transplantation of microbiota from ill donors to healthy recipients. In contrast, the symptoms and behaviours of depression and anxiety could also be transmitted from patients to healthy recipients through GM transfer. This evidence demonstrates the bidirectional role of the GM as a vector in disease.

The above results indicate that the microbiota-gut-brain axis plays an important pivotal role in the pathogenesis of depression, and its mechanism may be related to amino acid metabolism, the release of inflammatory factors, and the regulation of the HPA axis. In addition, the results suggest that the depression behaviours of depressed patients or animals can be transmitted to other healthy individuals through the GM, which provides a new means for the prevention and treatment of depression in the clinic

## Autism Spectrum Disorders

Autism spectrum disorders (ASDs) are neurodevelopmental disorders characterized by impairments in communication and social interactions along with restrictive and repetitive behaviours. Studies have shown that GM and its metabolites are not only related to GI problems but also linked to the behavioural symptoms of ASD (Svoboda, 2020).

In one study, GM of ASD patients or typically developing individuals were transplanted into GF mice, and the offspring of mice colonized with ASD GM showed autistic behaviour (Sharon et al., 2019). The GM of autistic patients and ASD mice were significantly different from that of the control group. In the GM of autistic patients/mice, *Lachnospiraceae* was increased significantly, while *Bacteroides ovatus* and *Parabacteroides merdae* were decreased significantly. Spearman’s rank correlation analysis showed that *Bacteroides ovatus* and *Parabacteroides merdae* were positively correlated with a decrease in repetitive behaviours and an increase in social behaviours, while the species of *Lachnospiraceae* were negatively correlated. A study found that a maternal high-fat diet (MHFD) induced behavioural alterations in offspring, the GM of which was characterized by a significant reduction in *Lactobacillus reuteri* (*L. reuteri*). In response to this phenomenon, researchers treated autistic mice with *L. reuteri* through intragastric administration, and the results showed that *L. reuteri* treatment selectively reversed ASD-like social deficits in genetic, environmental and idiopathic models of ASD (Sgritta et al., 2019). The mechanism may be the restoration of social-interaction-induced synaptic plasticity in the ventral tegmental area of the midbrain through the vagus nerve to improve the social deficits of mice.

In addition to depression and ASDs being closely related to GM disorders, studies have shown that GM disorders are found in patients with schizophrenia, Alzheimer’s disease, bipolar disorder, and other mental illnesses. A study showed that the transplantation of the GM from schizophrenic patients could cause schizophrenia-like symptoms such as psychomotor hyperactivity and cognitive impairment in antibiotic-treated mice (Zhu et al., 2019). The mechanism may be the relationship of these symptoms with abnormal tryptophan metabolism (the upregulation of the tryptophan-kynurenine-kynurenic acid pathway) caused by GM disorder. In terms of treatment, the symptoms of mice with Alzheimer’s disease or Parkinson’s disease were found to be improved through transplantation with GM from normal mice(Sun et al., 2018; Kim et al., 2020).

## Discussion

NCDs mainly include cardiovascular diseases (such as cardiopathy and stroke), cancer, chronic respiratory diseases, and diabetes. With the changes in lifestyle and the acceleration of population ageing, NCDs account for 71% of global deaths, causing 41 million deaths every year, and the impact on low-income and middle-income countries is particularly severe. Children, adults and elderly individuals are all susceptible to the risk factors for NCDs, while smoking, alcoholism, a lack of exercise and an inadequate diet will increase the risk of illness.

**Figure 2:**
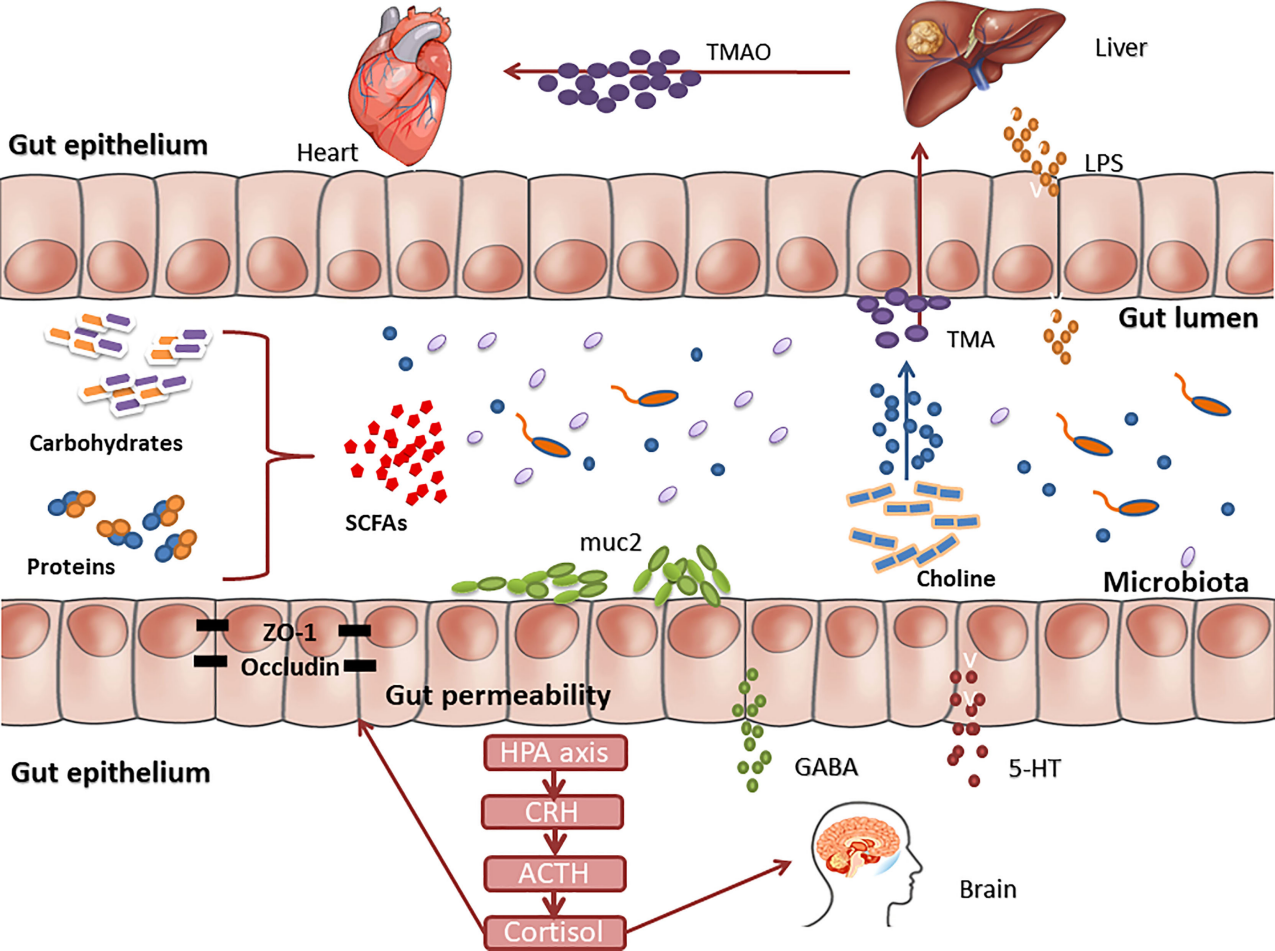
There are multiple pathways of communication between gut microbes and human organs.TMAO is generated via a metaorganismal pathway that begins with gut microbial conversion of dietary phosphatidyl choline into trimethylamine, followed by host liver oxidation to TMAO by flavin monooxygenases. Gut-liver axis, linking liver metabolism and gut microbiota, played an important role in the mechanisms of liver disease. Translocated LPS enters the liver through the hepatic portal vein and causes damage to liver function. Changes in the gut microbiota can affect the function of the central nervous system through multiple signaling pathways, including the hypothalamus-pituitary-adrenal axis, immune regulation, serotonin metabolism, and production of neuroactive compounds.

**Figure 3:**
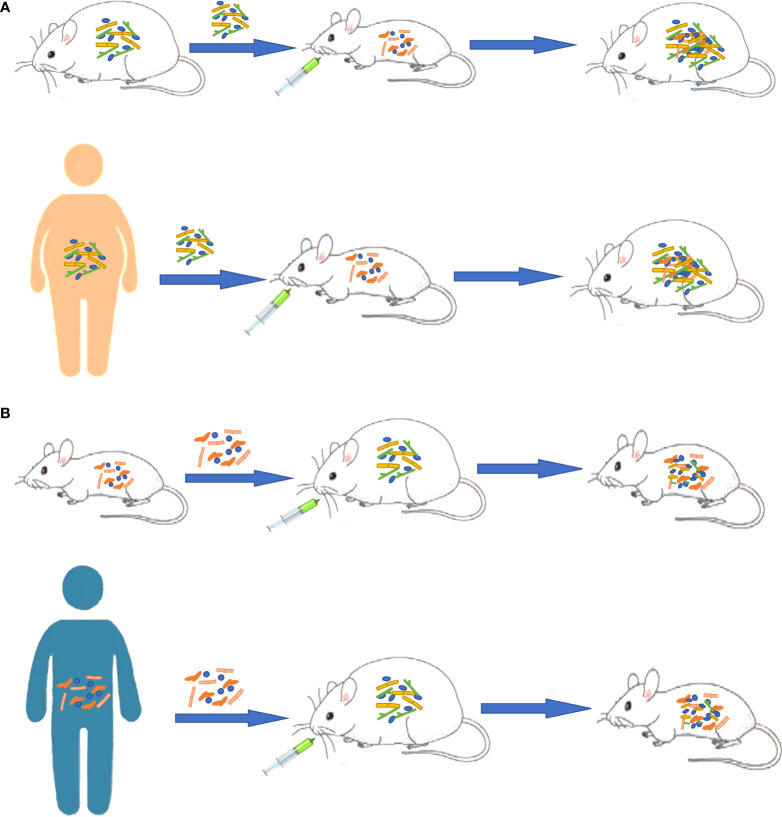
Different functions of gut microbiota as a vector in obesity. **(A)** Transplanting the gut microbiota of sick animals/people into GF mice or normal mice can replicate the disease phenotype. **(B)** Transplanting normal gut microbiota into sick animals can have an effect on treatment.

Increasing evidence shows that there is a strong correlation between GM disorders and the occurrence and development of various chronic NCDs (**Figure 2**). Through the above review, we found the following situations: (1) The transplantation of the flora of sick animals/individuals into GF mice or normal mice can replicate the disease phenotype, and correspondingly, the transplantation of the flora of healthy mice/individuals into sick mice/individuals can have a treatment effect (**Figure 3**). (2) Further research can determine the differential strains between individuals with diseases and healthy individuals (including pathogenic strains with high abundance in individuals with diseases or beneficial strains with a sharp decrease in abundance in individuals with diseases). The transplantation of pathogenic strains into GF mice can induce diseases, while the transplantation of beneficial strains into sick mice can alleviate the development of the disease and exert treatment effects.

The GM plays a dual role in chronic NCDs, and as a source of infection, a disordered GM can mediate the occurrence and development of diseases through FMT or the colonization of single pathogenic bacteria. Koch’s postulates also apply to the spread of NCDs among people. Taking diabetes as an example, patients with diabetes have GM disorders, and the pathogenic strains can be isolated from these patients and cultured in the laboratory. After the colonization of the gut of healthy individuals with these pathogenic strains, abnormal metabolic indicators appear, which leads to the occurrence of diabetes. Finally, the pathogenic strains can be isolated from the colonized individuals again. As a whole, a disordered GM acts as a source of infection, which may mediate the occurrence of some chronic NCDs and aggravate the diseases. Although it is a chronic transmission process, it still deserves our attention. In daily life, family and friends can pay attention to maintaining good hand hygiene habits and advocate the use of a separate diet, enhancing one’s physique, and maintaining good living habits. More attention should be given to the adjustment of the GM among patients to reduce the spread of undesirable GM among patients and prevent the resulting disease progression. From another point of view, the transplantation of a normal GM or key beneficial strains related to diseases can alleviate diseases and improve prognosis. The transplantation of whole flora or single probiotics to increase the beneficial metabolites produced by these strains provides a new idea for the treatment of GM disorder-related diseases. Faecal microbiota transplantation has been used in the treatment of IBS, IBDs and other intestinal diseases, but its long-term efficacy still needs large-sample RCT evidence (Xiang et al., 2020; Marcella et al., 2021).

A report suggests that chronic diseases in the traditional sense are communicable, and the findings from this review further support this point of view (Finlay, 2020). In the transfer of a pathogenic effect from a diseased individual to a healthy individual or of a therapeutic effect from a healthy individual to a diseased individual, the GM is critical as a transmission medium.

The mechanism involves immune system stress, amino acid metabolism, protein expression, and gene regulation. Focusing on the key node of GM can prevent the spread of diseases among people and explore new methods to treat diseases by intervening in the pathogenic links with the GM.

## Author Contributions

All authors have read and approved the manuscript. Study selection was completed by CC, XMY, and ZL. LZ, FJ and YL assessed the data for potential analysis. FB and XRY wrote a first draft of the text, while all authors contributed to subsequent drafts. FB facilitated article submission. All authors contributed to the article and approved the submitted version.

## Funding

This study was supported by the National Natural Science Foundation of China (Grant No.81873309), the Top academic talents of Jiangsu Provincial Hospital of Traditional Chinese Medicine (y2018rc06). This study was also supported by the National Natural Science Youth Fund (81904204).

## Conflict of Interest

The authors declare that the research was conducted in the absence of any commercial or financial relationships that could be construed as a potential conflict of interest.

## Publisher’s Note

All claims expressed in this article are solely those of the authors and do not necessarily represent those of their affiliated organizations, or those of the publisher, the editors and the reviewers. Any product that may be evaluated in this article, or claim that may be made by its manufacturer, is not guaranteed or endorsed by the publisher.

## Abbreviations

NCDs, Noncommunicable diseases; COVID-19, The Novel Coronavirus; GM, gut microbiota; SNPs, single nucleotide polymorphisms; T2DM, Type 2 diabetes mellitus; RYGB, Roux-en-Y gastric bypass, GF, germ-free; NAFLD, non-alcoholic fatty liver diseases; FMT, fecal microbiota transplantation; GABA, γ-aminobutyric acid; WEGL, Ganoderma lucidum mycelium; HSM, Hirsutella Sinensis; PCOS, Polycystic Ovary Syndrome; CVD, Cardiovascular disease; MBP, mean blood pressure; FTG, fecal transplant gavage; TMAO, Trimethylamine N-oxide; TMA, trimethylamine alpha; PPARα, peroxisome proliferator-activated receptor; NASH, nonalcoholic steatohepatitis; NAFLD, non-alcoholic fatty liver disease; FGIDs, Functional gastrointestinal diseases; DGBI, gut-brain interaction; FC, functional constipation; GI, gastrointestinal; 5-HT, 5-hydroxytryptamine; SERT, serotonin transporter; RCT, randomized controlled trial; STC, slow-transit constipation; IBS, Irritable bowel syndrome; IBS-D, IBS patients with diarrhea; IBD, Inflammatory bowel disease; UC, ulcerative colitis; CD, Crohn’s disease; CRC, Colorectal cancer; CMI, Cumulative methylation index; ACTH, adrenocorticotropic hormone; HPA, hypothalamic-pituitary-adrenal; CNS, central nervous system; LGG, L. rhamnosus; MDD, major depressive disorder; ASDs, Autism spectrum disorders; MHFD, Maternal high-fat diet; L. reuteri, Lactobacillus reuteri; CRH, corticotropin-releasing hormone; LPS, lipopolysaccharide; SCFAs, short-chain fatty acids.

## Glossary

NCDs: Noncommunicable diseases
COVID-19: The Novel Coronavirus
GM: gut microbiota
SNPs: single nucleotide polymorphisms
T2DM: Type 2 diabetes mellitus
RYGB: Roux-en-Y gastric bypass, GF, germ-free
NAFLD: non-alcoholic fatty liver diseases
FMT: fecal microbiota transplantation
GABA: γ-aminobutyric acid
WEGL: Ganoderma lucidum mycelium
HSM: Hirsutella Sinensis
PCOS: Polycystic Ovary Syndrome
CVD: Cardiovascular disease
MBP: mean blood pressure
FTG: fecal transplant gavage
TMAO: Trimethylamine N-oxide
TMA: trimethylamine alpha
PPARα: peroxisome proliferator-activated receptor
NASH: nonalcoholic steatohepatitis
NAFLD: non-alcoholic fatty liver disease
FGIDs: Functional gastrointestinal diseases
DGBI: gut-brain interaction
FC: functional constipation
GI: gastrointestinal
5-HT: 5-hydroxytryptamine
SERT: serotonin transporter
RCT: randomized controlled trial
STC: slow-transit constipation
IBS: Irritable bowel syndrome
IBS-D: IBS patients with diarrhea
IBD: Inflammatory bowel disease
UC: ulcerative colitis
CD: Crohn’s disease
CRC: Colorectal cancer
CMI: Cumulative methylation index
ACTH: adrenocorticotropic hormone
HPA: hypothalamic-pituitary-adrenal
CNS: central nervous system
LGG: L. rhamnosus
MDD: major depressive disorder
ASDs: Autism spectrum disorders
MHFD: Maternal high-fat diet
L. reuteri: Lactobacillus reuteri
CRH: corticotropin-releasing hormone
LPS: lipopolysaccharide
SCFAs: short-chain fatty acids.
